# Case Report: Primed dendritic cell immunotherapy for multiple solid tumors

**DOI:** 10.3389/fimmu.2026.1886898

**Published:** 2026-08-11

**Authors:** Jeffrey Jones, Aly Alrabaa, William Decker, Luis Ferbeyre, Gregorio Viramontes, Rafael Gonzalez, Elideth Angulo, Antonio Zamudio, Raul Hernandez, Stephen Noga, Susana Hernandez-Reyes, Paul Zhang, Darryl Smith, Ara Karamanian, Luis Tamara, Dalton Greenwood, Matthew Halpert

**Affiliations:** 1 Department of Urology or Immunology, Baylor College of Medicine, Houston, TX, United States; 2 Urology Section, Michael E DeBakey Veterans Affairs Medical Center, Houston, TX, United States; 3 Long School of Medicine, University of Texas Health Science Center San Antonio, San Antonio, TX, United States; 4 Immunocine Cancer Center, Cancún, Q.R., Mexico; 5 Onco Center Cancun, Cancún, Q.R., Mexico; 6 ReHealth, Cancún, Q.R., Mexico; 7 Amerimed Hospital, Cancún, Q.R., Mexico; 8 Oncology Department, University of Maryland Medical Center, Cantonsville, MD, United States; 9 Omega Precision Oncology, Houston, TX, United States; 10 HALO Precision Diagnostics (Prostate Laser Center), Houston, TX, United States

**Keywords:** cancer immunotherapy, dendritic cell therapy, metastatic melanoma, breast/ prostate adenocarcinoma, urothelial carcinoma

## Abstract

Many patients with advanced solid tumors ultimately develop resistance to conventional therapies, with especially limited options for those experiencing rapid progression or poor performance status, highlighting the need for personalized immunotherapies capable of inducing durable responses. In this retrospective case series, we describe the clinical course and outcomes of five adults (4 male, 1 female; median age 51 years [range, 39–62]) with metastatic solid tumors who achieved complete and durable remission following doubly loaded autologous dendritic cell therapy after failure of standard treatments, such as immune checkpoint inhibition, radiation, surgery, or endocrine therapy. Five patients, including prostate adenocarcinoma (n=1), malignant melanoma (n=2), triple-negative breast cancer (n=1), and urothelial carcinoma (n=1), were treated between July 2022 and March 2025 at a single-site referral-based immunotherapy center (Immunocine Cancer Center). Over the course of six weeks, patients received three vaccines of approximately 5–7 million modified dendritic cells injected adjacent to the lymph node draining to the tumor site. Follow-up ranged from 6 to 28 months, where radiographic response, clinical performance status, and tumor and available clinical and biological biomarkers were assessed. All five patients achieved complete radiographic remission confirmed by PET/CT or histology, with sustained responses ranging from 6 to 28 months and ongoing in all cases, and no grade 3 or higher adverse events observed. These observations support further evaluation of doubly-loaded autologous dendritic cell therapy in larger, controlled studies.

## 1 Introduction

Metastatic solid tumors remain a significant therapeutic challenge across cancer types, particularly when patients develop resistance to conventional treatments (1–3). Despite advancements in chemotherapy, targeted therapy, and immune checkpoint inhibitors (ICIs), many patients with advanced prostate adenocarcinoma, melanoma, triple-negative breast cancer (TNBC), or urothelial carcinoma ultimately experience disease progression and death (4).

Like fluorodeoxyglucose-positron emission tomography (FDG-PET) for other solid tumors, the emergence of prostate-specific membrane antigen-positron emission tomography/computed tomography (PSMA-PET/CT) for prostate cancer (PCa) has enabled earlier detection of oligometastatic recurrence, opening the door for metastasis-directed therapy (5, 6). Yet even with advanced imaging and focal interventions like stereotactic body radiation therapy, robotic surgical excision, or focal ablation, long-term disease control is typically elusive, especially when patients have exhausted standard-of-care (SOC) options such as androgen deprivation therapy (ADT), radiopharmaceuticals, and taxane-chemotherapy (7, 8).

Similarly, while immune checkpoint inhibitors have transformed the treatment landscape for metastatic melanoma, some patients demonstrate primary or acquired resistance, and overall response rates remain low for most indications (9, 10). Recent clinical studies have shown that combination checkpoint inhibitor therapy can extend overall survival to 15–20 months in some patients; yet, durable responses are limited and treatment failures common (11, 12). This is particularly devastating in those with high disease burden or rapid clinical decline. In breast and urothelial cancers, diseases for which response rates to immunotherapy remain modest, new strategies are urgently needed (13–15).

Chimeric antigen receptor T-cell (CAR-T) therapy represents a promising therapeutic development, although autoimmune-type toxicities may be dose-limiting (16). Moreover, to date, no CAR-T product targeting non-B-cell tumor types has been approved in the United States of America; however, China has approved a CAR-T therapy for gastric malignancies targeting Claudin18.2, a tight-junction protein that is highly overexpressed in gastrointestinal cancers while showing relatively restricted expression in healthy tissues (17). Although this is a significant breakthrough, the lack of appropriate and comprehensive cell surface targets on most solid tumor types remains a substantial limitation to the CAR-T approach.

In contrast, dendritic cell (DC) immunotherapy offers a highly personalized, more physiological approach by stimulating the adaptive immune system through direct tumor-specific antigen presentation (18). Existing DC-based strategies have used defined peptides, tumor lysates, or tumor-derived nucleic acids as antigen sources, with varying approaches to DC activation and maturation. For example, Sipuleucel-T utilizes autologous antigen-presenting cells activated ex vivo with a fusion protein composed of prostatic acid phosphatase and granulocyte-macrophage colony-stimulating factor (GM-CSF), thereby directing the immune response toward a shared prostate tumor antigen rather than the patient-specific tumor antigen repertoire (19). CreaVax-RCC instead sensitizes autologous DCs with tumor lysate and keyhole limpet hemocyanin, whereas APCEDEN utilizes whole-tumor lysate-loaded DCs matured with polyinosinic:polycytidylic acid (20–22). Although these strategies facilitate antigen presentation and DC activation, they are not expected to engage the multi-enzyme aminoacyl-tRNA synthetase (mARS) complex because they were not specifically designed to provide homologous antigenic information simultaneously to the major histocompatibility complex (MHC) class I and II pathways.

Recent mechanistic discoveries suggest that simultaneous presentation of homologous MHC class I and class II-associated antigenic sequences may engage the mARS complex, providing an additional mechanism for DC-mediated immune polarization (23–26). This 1.5-MDa complex within the endosomes of the DC compares peptide sequences associated with MHC class I and II compartments. When sufficient antigenic homology is detected, conformational changes within the mARS complex promote the release of aminoacyl-tRNA synthetase complex-interacting multifunctional protein 1 (AIMP1/p43), a mediator required for interleukin 12 production and downstream T helper 1 (Th1) polarization in experimental models (25). This mechanistic framework provides the rationale for homologous “double-loading,” in which DCs are supplied with patient-specific tumor mRNA and tumor lysate to provide overlapping antigenic information through MHC class I and II pathways. By contrast with single-antigen or single-source antigen-loading strategies, double loading is designed to engage this mARS-associated pathway and promote a broader, Th1-based antitumor immune response (25, 27–31). Clinical trials of double-loaded DCs for aggressive solid malignancies, including glioblastoma (NCT04552886) and pancreatic ductal adenocarcinoma (NCT04157127), have reported encouraging preliminary findings (32).

All patients in this case series received double-loaded DC therapy through a standardized three-phase Immunocine™ Dendritic Cell therapy (IDCT) protocol outside the framework of formal clinical trials. The patients first received 5 micrograms/kg of granulocyte colony-stimulating factor (G-CSF), filgrastim subcutaneously daily over five consecutive days to mobilize white blood cells, concurrent with biopsy to harvest tumor antigens. Apheresis was then performed to collect peripheral blood mononuclear cells for DC production. Monocytes were isolated and differentiated ex-vivo into DCs using a combination of 0.5 ml of GM-CSF stock solution at 2ug/mL (Miltenyi 170-076-136), for a final media concentration 20 ng/ml, and 0.5 mL of Interleukin 4 (IL-4) stock solution at 1ug/mL (Miltenyi 170-076-135), resulting in a final media concentration of 10 ng/ml. Cells were cultured in a final total volume of 50 mL CTS AIM-V media supplemented with 10% inactivated autologous plasma media per T225 flask over a 6-day incubation period. The resulting DCs were then “double-loaded” with patient-specific tumor mRNA and lysate matured with a proinflammatory cytokine cocktail by the method of Decker et al. (2006), enabling tumor-specific antigen presentation via both MHC class I and II pathways (34). Total tumor mRNA was amplified according to the method of Slagter-Jager et al, with technical modifications to enhance fidelity and reproducibility (33). Final DC products underwent in-house and external quality-control assessment. For cell enumeration and dosing determination, acridine orange/propidium iodide fluorescence was measured using a LUNA™ Stem automated cell counter (Logos Biosystems). Cell viability was additionally assessed by 7-aminoactinomycin D staining during flow cytometric analysis, while DC identity was evaluated primarily by CD11c and MHC class II (HLA-DR) expression. Additional phenotypic markers and patient-specific viability results are provided in **Supplementary Table 1**. Aliquots of the final DC products were also submitted for independent flow cytometric validation to Diagnóstico Molecular de Leucemias y Terapias Celulares, a laboratory accredited by the Entidad Mexicana de Acreditación. Antigen loading was assessed by direct sequencing of the final cellular product. Each injection contained approximately 5–7 million mature DCs, with individual doses ranging from 1.5-20.6 million cells; patient-specific cell yields and administered doses are provided in **Supplementary Table 2**. Patients then received three image-guided perilymphatic injections every two weeks over the course of six weeks, typically adjacent to lymph nodes most adjacent to the tumor site. 180 micrograms of pegylated interferon-alpha (IFN2a) was co-administered intramuscularly with each IDCT injection in either the right or left deltoid throughout the six-week treatment period. Some patients also continued systemic therapy, such as tamoxifen or checkpoint inhibitors, concurrent with the immunotherapy protocol. Informed consent was obtained from all of the patients for treatment and for publication of this case series, including all accompanying images. Ethics approval was granted by COFEPRIS (Comisión Federal para la Protección contra Riesgos Sanitarios; Federal Committee for Protection from Sanitary Risks) based on FDA clinical trials for other tumor histologies (Clinical Trial # NCT04157127, Clinical Trial Date 2020-08-03).

This first-time report case series presents five patients with metastatic solid tumors, all having failed standard therapies, who subsequently experienced a complete response following treatment with a doubly-loaded autologous DC therapy. The approach incorporates advances in DC preparation utilizing antigen loading and administration techniques that have shown promise in other malignancies, but have not been previously reported in metastatic prostate, melanoma, breast, or urothelial carcinoma (34–36). These five cases are drawn from the first 80 patients, 82% of whom had stage IV cancer after failing at least one line of SOC therapy. In this cohort, an objective response rate of 74% was observed, and 39% of patients achieved either no evidence of disease (NED) or long-term stability (>2 years).

## 2 Case reports and results

### 2.1 Prostate adenocarcinoma

A 62-year-old White male with no smoking history and only occasional alcohol use underwent robot-assisted radical prostatectomy with extended pelvic lymph node dissection for PCa in September 2019. Pathology revealed intraductal adenocarcinoma, Gleason 4 + 3 with 10% tertiary pattern 5, extensive left neurovascular bundle extra-prostatic extension, bilateral seminal vesicle invasion and bilateral microscopic pelvic nodal involvement, 3 nodes-left, 2-right. Postoperative prostate-specific antigen (PSA) declined to 0.04 ng/mL but rose to 0.06 ng/mL two months later. Imaging was initially negative. The patient underwent adjuvant pelvic proton radiation at 6 months post-op, and after eighteen months of ADT exhibited undetectable PSA.

Six months later, while still on ADT, the patient’s PSA rose to 0.53 ng/mL. Castrate-resistant PCa recurred sequentially in multiple areas: the right perirectal side wall followed by a sub-diaphragmatic implant, a common iliac lymph node, and a substernal lymph node. All were managed with focal ablation, using laser, cryotherapy, or irreversible electroporation. Each time the oligo-metastatic lesions were successfully ablated as determined by both PSMA-PET/CT scanning and magnetic resonance imaging (MRI), however, the PSA did not decline substantially, despite combination ADT + androgen receptor inhibitor. Chemotherapy was considered but was withheld due to COVID pandemic-era concerns for immune compromise.

**Figure 1:**
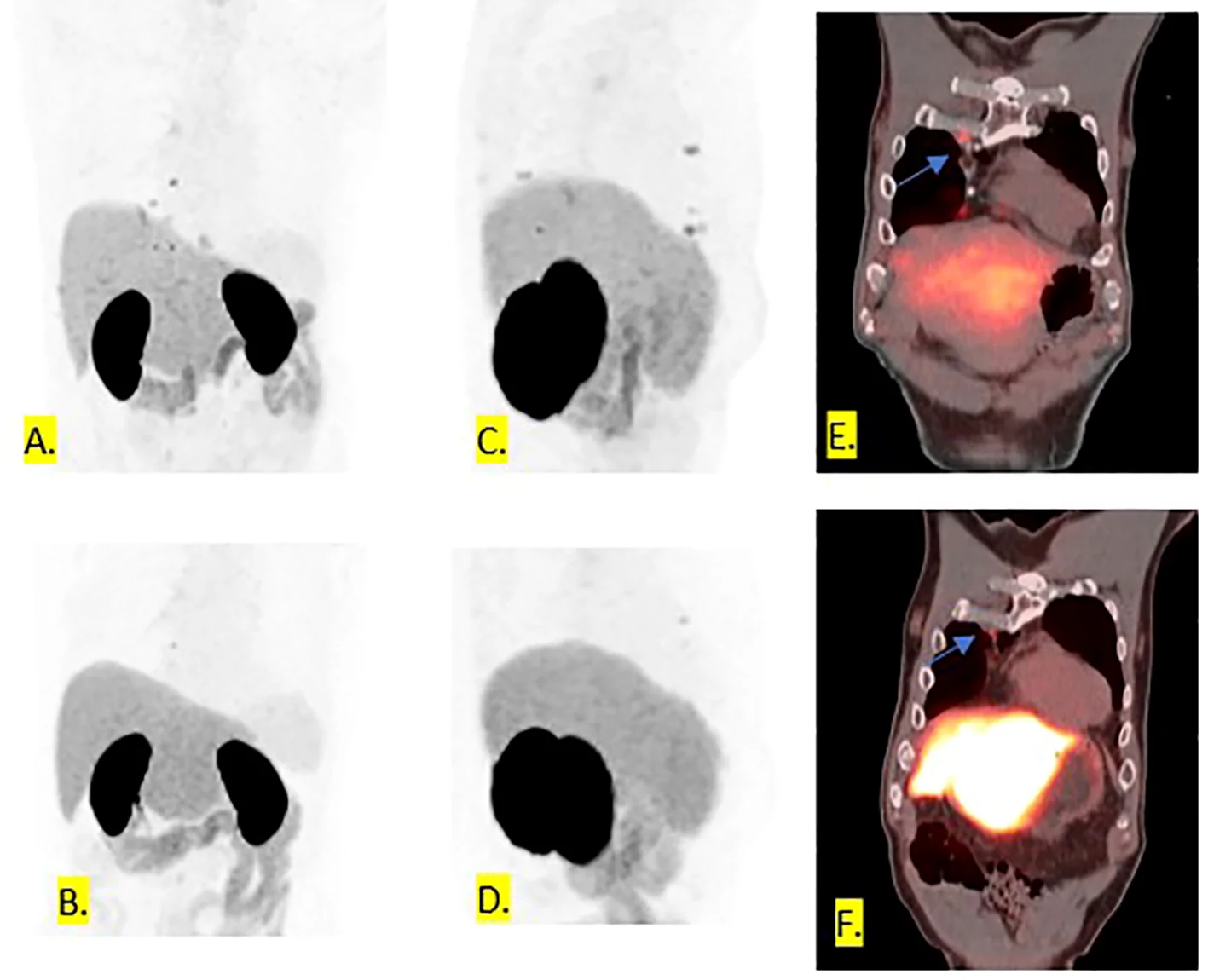
Pre- and post-treatment psma-pet/ct imaging in prostate adenocarcinoma. All images were obtained using the same scanner. Pre-treatment scans were performed 1 year apart pre and post IDCT. **(A)** Coronal MIP image showing multiple PSMA-avid thoracic lymph nodes prior to IDCT, at which time PSA was 78 ng/ml. **(B)** Coronal MIP image obtained two months after the third IDCT injection, demonstrating resolution of all lesions except one. **(C)** Sagittal MIP image prior to IDCT. **(D)** Sagittal MIP image following IDCT. **(E)** Coronal fusion image prior to treatment, with a standardized uptake value (SUV) of 3.1 at the pointed internal mammary lymph node mass. **(F)** Coronal fusion image post-treatment, showing reduced SUV of 1.5 of the one remaining internal mammary lymph node (pointed).

Eleven months later, PSA rose to 73 ng/mL and PSMA-PET/CT revealed multiple mediastinal metastases, verified on MRI with gadolinium (**Figures 1A, C, E**). Circulating tumor cell (CTC) count was 4/mL and the patient’s PSA peaked at 140 ng/mL. Treatment with a combination of Po Salicinum plus topical and sub-lingual Gc protein-derived macrophage activating factor was used while the patient was being qualified for IDCT. Two months later, following the initial IDCT injection to the right inguinal region adjacent to the right inguinal lymph nodes, the patient’s PSA declined to 7.8 ng/mL. PSA subsequently decreased to 2.5 ng/mL after the second injection to the bilateral inguinal regions, to 0.7 ng/mL before the third injection, and to 0.6 ng/mL following the third injection in the bilateral axillary region adjacent to the axillary lymph nodes. The prognostic WREN PROSTest, a machine-learning based, 27-gene qPCR liquid biopsy with a diagnostic cutoff of ≥50/100, returned 7/100, consistent with disease control (37, 38).

Treatment was well tolerated, with only minimal adverse events (AEs) of back pain during filgrastim and 24 hours of myalgias with IFN2a. Repeat PSMA-PET/CT showed resolution of all lesions (**Figures 1B, D, F**) except one faint internal mammary lymph node (**Figure 1F**), which was cryo-ablated. At the six-month follow-up, the patient remains in near-complete remission, without biochemical evidence of progression.

### 2.2 Melanoma

A 39-year-old White male with a history of cutaneous melanoma, previously treated with complete surgical resection with negative margins, presented with progressive respiratory symptoms including wheezing and dyspnea. He had a prior history of smoking and alcohol use, but reported cessation of both at the time of evaluation. Chest X-ray revealed a lower left lobe mass, and a computed tomography (CT)-guided biopsy confirmed metastatic melanoma with immunohistochemistry positive for SOX10 and focal S100, consistent with melanocytic origin.

**Figure 2:**
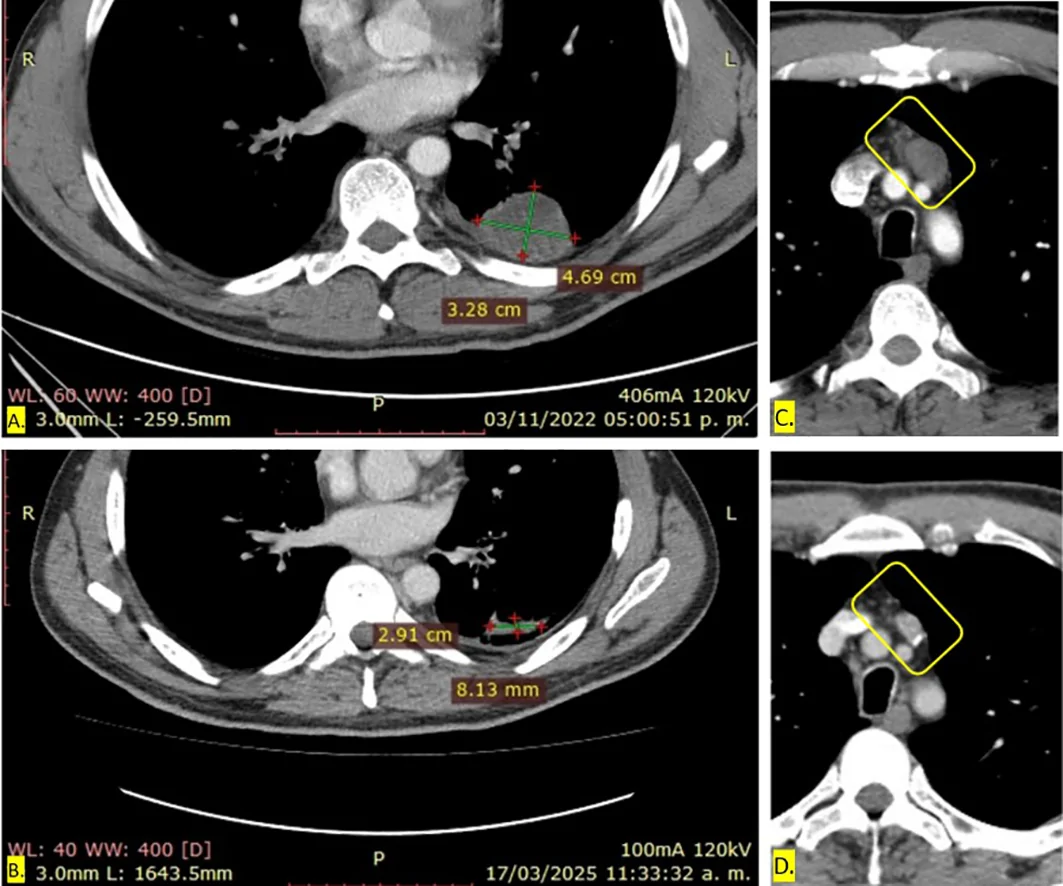
CT imaging demonstrating response to treatment in malignant melanoma. **(A)** Baseline (Nov 2022) contrast-enhanced CT shows a 47 × 33 mm lung mass selected as the index lesion for response assessment. **(B)** CT scan at 27 months (March 2025) post-treatment shows evolution to a flat, soft tissue density measuring 29×8mm, consistent with post-treatment fibrosis. **(C)** Baseline CT showing enlarged mediastinal lymphadenopathy (outlined in yellow). **(D)** CT at 27-month follow-up showing marked reduction in the previously identified lymph node (outlined in yellow) with dense, calcified appearance and peripheral calcification. The transformation from bulky, soft tissue density lymphadenopathy to small, dense, calcified residual tissue represents complete treatment response with fibrotic replacement of previously active disease.

Staging CT scans 6 months later revealed a left lower lobe mass of 4.69cm in greatest dimension with satellite nodular opacities along the major fissure, scattered nodular pleural thickening with extension into paraspinal soft tissues at T10, and mediastinal/hilar lymphadenopathy (**Figures 2A, C**). CT of abdomen, pelvis, and neck revealed left supraclavicular lymphadenopathy, but no other metastases.

The patient received checkpoint inhibitor therapy with ipilimumab (3mg/kg) and nivolumab (1mg/kg) every three weeks for two cycles, yet experienced rapid clinical deterioration from Eastern Cooperative Oncology Group (ECOG) 0 to ECOG 3–4 over a four-week period. Repeat imaging confirmed progressive disease with a 29% increase in the primary left lower lobe mass, from 4.69 to 5.8 cm, and worsening lymphadenopathy across multiple sites indicating primary ICI resistance.

Two months later, the patient received IDCT injections to the left supraclavicular region adjacent to the left supraclavicular lymph nodes. There were minimal AEs, such as mild fatigue, diarrhea, and changes in hair color, however, no AE required discontinuation of therapy. CT post-treatment revealed a reduction of primary lung mass to 2.9 cm, and no new lesions were identified (**Figure 2B, D**). Multiple biopsies confirmed the absence of viable tumor. A month following treatment, the patient demonstrated an ECOG 0 status with resumption of modified physical activities, and three to five months following treatment, patient regained pre-diagnosis activity levels.

### 2.3 Parallel case: desmoplastic melanoma

**Figure 3:**
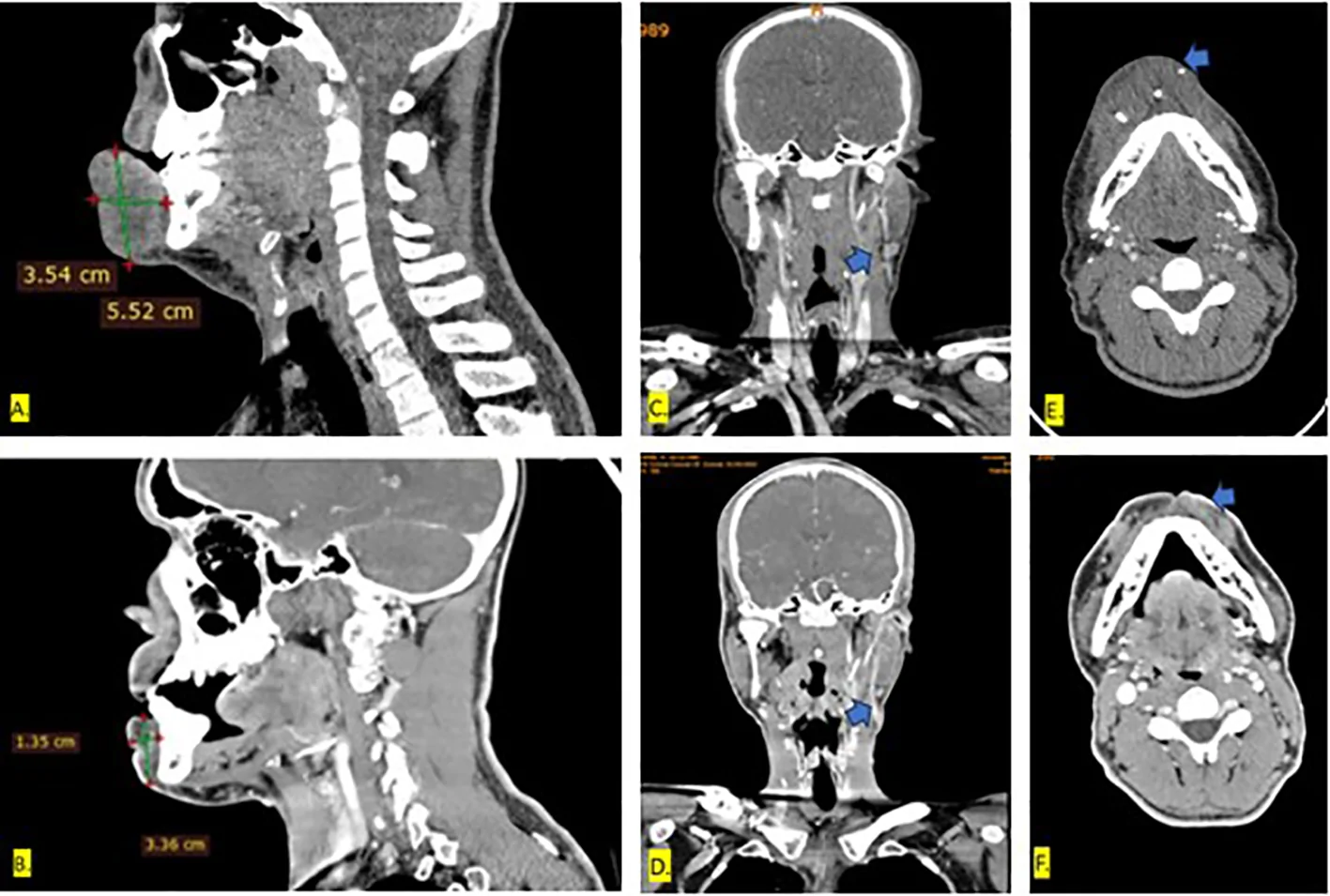
CT imaging demonstrating response to treatment in desmoplastic melanoma. **(A)** Baseline imaging prior to IDCT initiation demonstrating a large heterogeneous mass involving the lower lip and anterior mandibular region measuring 5.52×3.54 cm. **(B)** Six months following treatment initiation showing complete resolution of the previously identified mass with restoration of normal lip contour. The lower lip appears flat with normal soft tissue density and no evidence of residual tumor. **(C)** Pre-IDCT Coronal CT of the head and neck shows a cervical lymph node with ovoid morphology in the left parotid space and ipsilateral level IV cervical region (pointed). **(D)** Post-treatment image shows decreased lymph node size (pointed). **(E)** Pre-treatment Axial CT of the lower lip and chin demonstrates an ovoid soft tissue mass with heterogeneous density, hypodense center, peripheral soft tissue density, and mild peripheral enhancement (pointed). **(F)** Post-treatment image shows interval reduction in mass size (pointed).

A 33-year-old White male with no history of smoking or alcohol use underwent a sub-total resection of a desmoplastic melanoma of the lower lip, bilateral neck dissection, and reconstruction using a radial forearm free flap. Pathology revealed an 11mm desmoplastic melanoma ulceration, neurotropism, and 11 mitoses/mm^2^. Within six months, despite adjuvant pembrolizumab, the patient experienced local recurrence with tumor involving almost the entire reconstructed lip flap, indicative of acquired ICI resistance. Imaging showed a 4.9×2.9×5.6cm mass with mandibular periosteal involvement and cervical lymphadenopathy (**Figures 3A, C, E**).

Five months after receiving IDCT injections to the neck adjacent to the cervical lymph nodes, imaging documented mass reduction to 3.36×1.35×2.4cm with complete lymph node resolution (**Figures 3B, D, F**). 18 months post-treatment, the patient ceased ICI and fully resumed all activities and personal pursuits. Over 28 months post-treatment, the most recent imaging studies show that the patient remains in complete remission with NED.

### 2.4 Triple-negative breast cancer

A 58-year-old White female, with a 12-pack-year smoking history but reported cessation of 10 years at the time of evaluation, who had been diagnosed with TNBC in 2021 and had been treated with anthracycline+taxane-based chemotherapy, underwent lumpectomy and right axillary lymph node dissection revealing 11/12 positive nodes in 2022. Pathology from the mass excised from the right breast revealed recurrent invasive ductal carcinoma, Nottingham grade 3 (3 + 3 + 2), with margin involvement (≥12 mm) and lymphovascular invasion. Thus, the patient received adjuvant radiotherapy and several rounds of platinum-based chemotherapy.

In July 2024, staging workup demonstrated hypermetabolic right axillary/subpectoral and right cervical (5B) nodes (SUVmax 2.9-4.1) on PET/CT (images not shown). Fine-needle aspiration of the right cervical node confirmed metastatic adenocarcinoma, SOX10+/CKAE1/3+, ER/PR/HER2 negative, AR negative. Baseline laboratory results demonstrated lactate dehydrogenase (LDH) 293 U/L, D-dimer markedly elevated, C-reactive protein (CRP) and erythrocyte sedimentation rate (ESR) elevated, and neutrophil-to-lymphocyte ratio (NLR) ~5.0. Traditional tumor markers (CA 27-29, CA 15-3) remained within normal ranges and a Northstar genomic assay showed a methylation score of 370, consistent with high tumor burden.

In September 2024, the patient initiated IDCT therapy to the inguinal region adjacent to the inguinal lymph nodes. Serial labs demonstrated significant biologic response: LDH declined to 193 U/L, NLR improved to 2.4, CRP and ESR normalized, and CA 27–29 remained stable. At six-month follow-up, the patient reported ECOG 0–1 performance status and preserved quality of life. Objective markers indicated metabolic and immune improvement, despite tumor markers remaining uninformative.

By September 2025, the patient achieved complete remission with negative PET/CT scans (images not shown). LDH normalized, CA 27–29 and CA 15–3 remained stable within normal limits, and blood counts/metabolic panels were preserved. The patient remains ECOG 0 performance status with an excellent quality of life and NED, with no adjuvant chemotherapy or ICI.

### 2.5 Urothelial carcinoma

A 62-year-old White male with no history of tobacco or alcohol use and a complex oncologic history presented with two concurrent malignancies: metastatic urothelial cancer-bladder primary and multiple myeloma in remission for three years after autologous hematopoietic stem cell transplantation followed by daratumumab maintenance therapy.

During hospitalization for colitis, the patient was found to have microhematuria on cystoscopy, and transurethral resection of bladder tumor revealed invasive high-grade, T2b urothelial carcinoma of the bladder, with a high proliferation index (Ki-67 at 90%). FDG-PET/CT showed no evidence of FDG-avid metastatic disease; thus, the patient opted for bladder-preservation and chemotherapy rather than a radical cystectomy.

**Figure 4:**
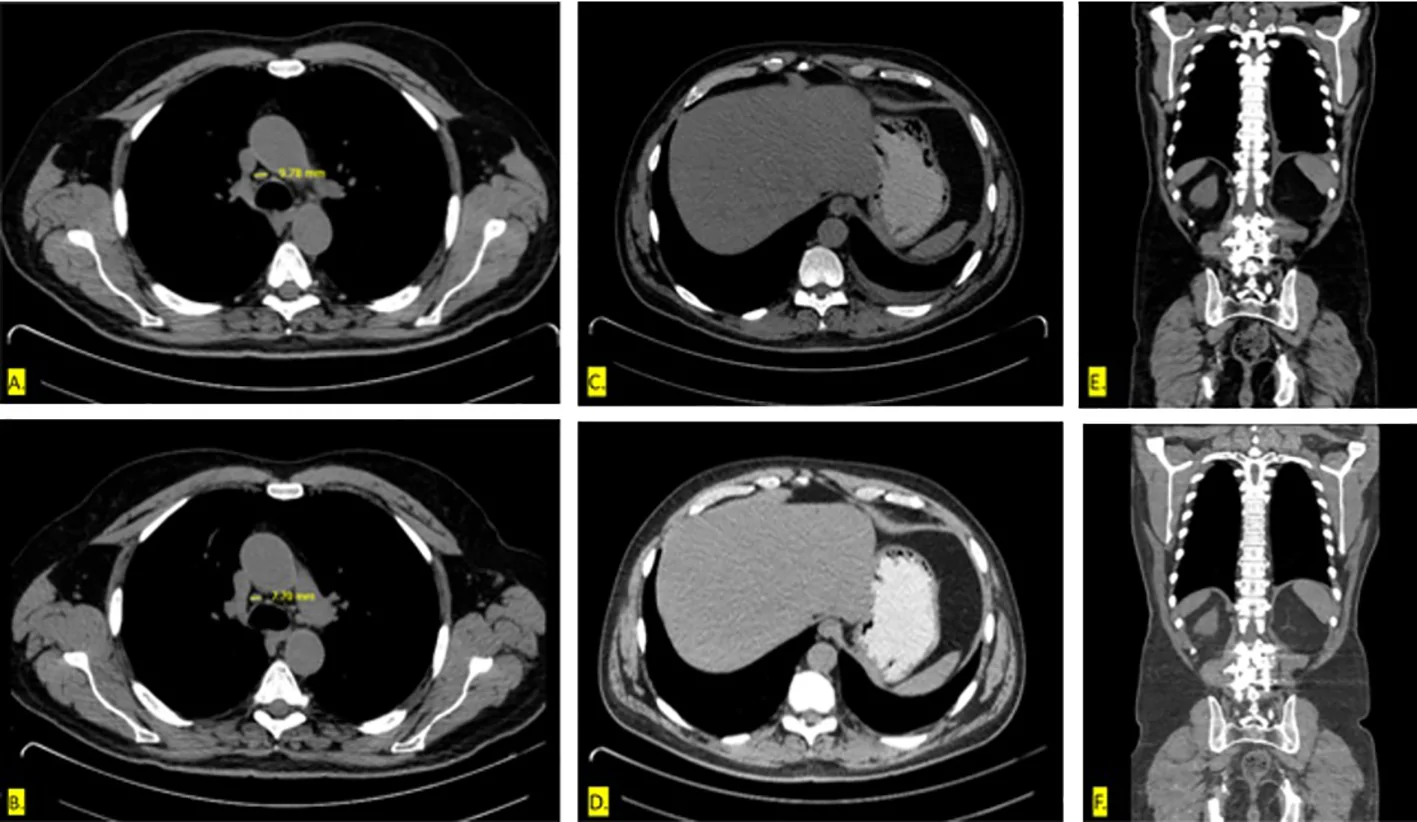
Pre- and post-treatment ct imaging of hilar lymph node and pleural effusion. **(A)** Axial CT of the chest prior to IDCT demonstrates an enlarged hilar lymph node measuring 9.78mm in greatest diameter. **(B)** Corresponding post-IDCT axial CT reveals interval reduction in size of the same lymph node to 7.7mm, without evidence of new lymphadenopathy or pathological enhancement. **(C)** Axial CT of the chest prior to IDCT demonstrates left-sided pleural effusion (pointed) with associated pleural thickening. **(D)** Axial chest CT post-IDCT shows complete resolution of the effusion and pleural changes (pointed). **(E)** Coronal chest CT pre-IDCT provides an additional view of the left pleural effusion (pointed). **(F)** Coronal chest CT following IDCT demonstrates normalization of the pleural space (pointed).

Four months later, the patient experienced disease progression and clinical deterioration. FDG-PET/CT demonstrated aggressive metastatic spread with multiple left hilar lymph node metastases-the largest measuring 2.4×1.6cm with SUV of 6.6, malignant left pleural effusion, and a left pleural lesion (**Figures 4A, C, E**). A pleural catheter was placed to drain the effusion due to dyspnea. Laboratory studies revealed severe anemia (hemoglobin 8.2g/dL, red blood cell (RBC) 2.59million/µL, hematocrit 26.5%, mean corpuscular volume 102.3fL) with nucleated RBCs. Liver function rests showed significant dysfunction, with low albumin (2.2g/dL), elevated alkaline phosphatase (299U/L), and transaminases (ALT 118U/L, AST 80U/L).

Faced with limited SOC options, the patient underwent IDCT, receiving three injections to the right inguinal region adjacent to the right inguinal lymph nodes. Imaging three months post-treatment revealed complete resolution of the pleural effusion and lymphadenopathy (**Figures 4B, D, F**). At 18-month follow-up, the patient remained in complete remission with NED on cross-sectional imaging or cystoscopic examination. The patient’s physical condition and quality of life have significantly improved, and the patient has returned to normal daily activities. Treatment-related AEs were minimal (grade 1-2), with patient experiencing only mild flu-like symptoms after the first injection and transient fatigue.

## 3 Discussion

This case series demonstrates a pattern of complete and short- to intermediate-duration responses to IDCT across four distinct metastatic solid tumors: prostate adenocarcinoma, malignant melanoma, TNBC, and urothelial carcinoma. All patients received available conventional therapies, yet each experienced recurrence or progression to advanced stage disease. After exhausting all SOC options, each patient achieved clinically complete, or near-complete, remission with IDCT.

In contrast to conventional treatments for metastatic cancer, which often carry significant toxicity profiles that heavily impact patients’ quality of life, IDCT was well-tolerated in all cases. For example, immune checkpoint inhibitors commonly induce thyroid dysfunction, with a median onset of approximately 5.3 weeks following treatment initiation (39). Following treatment, the patient with PCa remains in biochemical remission at 6 months. The patients with melanomas have sustained complete remission well beyond two years, well exceeding the 6-month survival for patients with poor ECOG performance and high disease burden (10). The breast and bladder cancer patients have remained in complete remission for 12–18 months, far longer than the historical benchmarks for endocrine or salvage therapy in these settings (40). These timeframes are significant because durable responses in stage 4 disease, especially in checkpoint-refractory, chemotherapy-refractory, radiotherapy-refractory, hormone-refractory, or heavily pretreated patients, are uncommon outside of select immunotherapy responders (41).

Each case in this case series carried clinicopathological features typically associated with poor responsiveness to immunotherapy. The PCa case had castration-resistant disease, with low mutational burden, high tumor burden, and androgen receptor-independent clones that are known to acquire immune escape mechanisms (41, 42). The melanoma cases had both forms of checkpoint inhibitor failure- one demonstrating primary resistance, and the other acquired resistance during adjuvant therapy (12, 41). The breast cancer case involved heavily pretreated, poorly differentiated TNBC, a subtype often regarded as immunologically “cold” due to sparse tumor infiltrating lymphocytes (TILs), high systemic inflammation, and impaired antigen presentation (15, 43). The urothelial carcinoma case exhibited multiple immune-suppressive features, including visceral pleural metastases with malignant effusion, reduced functional status, concurrent multiple myeloma, and a history of immunosuppressive treatments including stem cell transplantation, radiation, and daratumumab maintenance (40, 44). Collectively, these features would be expected to confer a low likelihood of meaningful response to immunotherapy. The observation that each patient achieved complete or near-complete remission suggests that double-loaded DC therapy may be capable of overcoming diverse resistance mechanisms across tumor types, including immunologically “cold” microenvironments and checkpoint-refractory disease.

While ICIs have revolutionized the treatment of melanoma and some bladder and breast cancers, many patients experience primary or acquired resistance (9). In PCa, for which immunotherapy is less well established, standard management often includes radiation and ADT, but outcomes remain poor once metastases recur (42, 45). Similarly, patients with TNBC, who relapse after standard chemotherapy, often have limited therapeutic options, and although immune checkpoint inhibitors have shown some activity, most patients ultimately develop resistance and poor outcomes (43). Checkpoint inhibitors work by lifting immune suppression, but they require pre-existing TILs or endogenous tumor-specific T-cells to be effective (46). In “cold” tumors lacking TILs, the immune system may never have received the right signals to generate a response. By supplying activated, antigen-loaded DCs, the therapy may prime the immune system to recognize and attack tumor antigens, turning “cold” tumors hot and enabling checkpoint inhibitors to work when previously ineffective.

The therapy employed was built upon lessons learned from prior limitations in DC treatment design. Traditional DC therapies frequently underperform due to suboptimal antigen loading and lack of adequate costimulatory signals (4, 23). Previous DC therapy trials in solid tumors yielded limited success, often due to weak antigen presentation, poor T-cell activation, and unabated tumor-induced immunosuppression (47). Th1 may have been limited by failure to activate the mARS complex, promote AIMP1 release, and expand key effector subsets (29, 48). By contrast, the IDCT protocol employs dual antigen loading with tumor mRNA to facilitate MHC Class I presentation and tumor lysate to facilitate MHC Class II presentation, combined with ex-vivo DC maturation with cytokines, image-guided injection near tumor-draining lymph nodes, and adjuvant type I interferon. Combining DC vaccination with strategic injection near lymphatic drainage sites and type I interferon results in potent immune responses capable of reversing immune escape mechanisms and overcoming ICI resistance (49).

This case series is limited by its small, heterogeneous sample and retrospective observational design, precluding definitive conclusions regarding treatment efficacy. Its primary purpose was to demonstrate the potential applicability of this therapeutic approach across multiple tumor types rather than to establish efficacy within any single malignancy. Because treatment was administered outside traditional FDA-approved frameworks and all patients were sufficiently well to travel, the findings may also be subject to selection bias and limited generalizability. Although the outcomes are encouraging, larger prospective studies and well-designed clinical trials are needed to confirm these observations.

## 4 Conclusion

This case series describes complete or near-complete responses following IDCT in five patients with advanced, treatment-refractory solid tumors and provides preliminary observations supporting further evaluation of this therapeutic approach. Patients with metastatic prostate adenocarcinoma, malignant melanoma, TNBC, and urothelial carcinoma achieved prolonged disease control despite prior failure of multiple standard therapies and adverse immunologic prognostic features, including ICI resistance and immunosuppressive treatments.

Although these findings are observational, the consistency of responses, long-term remission, and stability across tumor types justifies further investigation of this approach. Controlled clinical trials are warranted to evaluate efficacy and define optimal combination strategies. In an era increasingly defined by precision oncology, this approach represents a compelling strategy for transforming immune-inert tumors into targets of effective immune attack and may offer new hope to patients with limited therapeutic options.

## Supplementary material

SUPPLEMENTARY TABLE 1 Specific dendritic cell identity and viability markers for the individual cases.

SUPPLEMENTARY TABLE 2 Additional information about the DC treatment.

## References

[1] BonottoM GerratanaL PolettoE DriolP GiangrecoM RussoS . Measures of outcome in metastatic breast cancer: insights from a real-world scenario. Oncologist. (2014) 19:608–15. doi: 10.1634/theoncologist.2014-0002 24794159 PMC4041678

[2] GershenwaldJE ScolyerRA HessKR SondakVK LongGV RossMI . Melanoma staging: evidence-based changes in the American Joint Committee on Cancer eighth edition cancer staging manual. CA Cancer J Clin. (2017) 67:472–92. doi: 10.3322/caac.21409 29028110 PMC5978683

[3] von der MaaseH HansenSW RobertsJT DogliottiL OliverT MooreMJ . Gemcitabine and cisplatin versus methotrexate, vinblastine, doxorubicin, and cisplatin in advanced or metastatic bladder cancer: results of a large, randomized, multinational, multicenter, phase III study. J Clin Oncol. (2000) 18:3068–77. doi: 10.1200/JCO.2000.18.17.3068 11001674

[4] BolKF SchreibeltG GerritsenWR de VriesIJM FigdorCG . Dendritic cell-based immunotherapy: state of the art and beyond. Clin Cancer Res. (2016) 22:1897–906. doi: 10.1158/1078-0432.ccr-15-1399 27084743

[5] HennrichU EderM . 68Ga]Ga-PSMA-11: The first FDA-approved 68Ga-radiopharmaceutical for PET imaging of prostate cancer. Pharm (Basel). (2021) 14:713. doi: 10.3390/ph14080713 34451810 PMC8401928

[6] ZhaoG JiB . Head-to-head comparison of 68Ga-PSMA-11 PET/CT and 99mTc-MDP bone scintigraphy for the detection of bone metastases in patients with prostate cancer: a meta-analysis [published correction appears in AJR Am J Roentgenol. 2022 Sep;219(3):529. doi: 10.2214/AJR.22.28240. AJR Am J Roentgenol. (2022) 219:386–95. doi: 10.2214/AJR.21.27323 35441529

[7] BhattasaliO ChenLN TongM LeiS CollinsBT KrishnanP . Rationale for stereotactic body radiation therapy in treating patients with oligometastatic hormone-naïve prostate cancer. Front Oncol. (2013) 3:293. doi: 10.3389/fonc.2013.00293 24350058 PMC3847811

[8] VialN ZilliT . Stereotactic body radiotherapy: a second chance for radio-recurrent prostate cancer. Prostate Cancer Prostatic Dis. (2025) 28:544–545. doi: 10.1038/s41391-025-00947-y 39915656

[9] PostowMA SidlowR HellmannMD . Immune-related adverse events associated with immune checkpoint blockade. N Engl J Med. (2018) 378:158–68. doi: 10.1056/NEJMra1703481 29320654

[10] SchadendorfD HodiFS RobertC WeberJS MargolinK HamidO . Pooled analysis of long-term survival data from phase II and phase III trials of ipilimumab in unresectable or metastatic melanoma. J Clin Oncol. (2015) 33:1889–94. doi: 10.1200/JCO.2014.56.2736 25667295 PMC5089162

[11] AsciertoPA McArthurGA . Checkpoint inhibitors in melanoma and early phase development in solid tumors: what's the future? J Transl Med. (2017) 15:173. doi: 10.1186/s12967-017-1278-5 28789707 PMC5549368

[12] RibasA WolchokJD . Cancer immunotherapy using checkpoint blockade. Science. (2018) 359:1350–5. doi: 10.1126/science.aar4060 29567705 PMC7391259

[13] EmensLA . Breast cancer immunotherapy: facts and hopes. Clin Cancer Res. (2018) 24:511–20. doi: 10.1158/1078-0432.CCR-16-3001 28801472 PMC5796849

[14] FradetY BellmuntJ VaughnDJ LeeJL FongL VogelzangNJ . Randomized phase III KEYNOTE-045 trial of pembrolizumab versus paclitaxel, docetaxel, or vinflunine in recurrent advanced urothelial cancer: results of >2 years of follow-up. Ann Oncol. (2019) 30:970–6. doi: 10.1093/annonc/mdz127 31050707 PMC6594457

[15] AdamsS Gatti-MaysME KalinskyK KordeLA SharonE Amiri-KordestaniL . Current landscape of immunotherapy in breast cancer: a review. JAMA Oncol. (2019) 5:1205–14. doi: 10.1001/jamaoncol.2018.7147 30973611 PMC8452050

[16] SternerRC SternerRM . CAR-T cell therapy: current limitations and potential strategies. Blood Cancer J. (2021) 11:69. doi: 10.1038/s41408-021-00459-7 33824268 PMC8024391

[17] QiC LiuC PengZ ZhangY WeiJ QiuW . Claudin-18 isoform 2-specific CAR T-cell therapy (satri-cel) versus treatment of physician's choice for previously treated advanced gastric or gastro-oesophageal junction cancer (CT041-ST-01): a randomised, open-label, phase 2 trial. Lancet. (2025) 405:2049–60. doi: 10.1016/S0140-6736(25)00860-8 40460847

[18] WculekSK CuetoFJ MujalAM MeleroI KrummelMF SanchoD . Dendritic cells in cancer immunology and immunotherapy. Nat Rev Immunol. (2020) 20:7–24. doi: 10.1038/s41577-019-0210-z 31467405

[19] KantoffPW HiganoCS ShoreND BergerER SmallEJ PensonDF . Sipuleucel-T immunotherapy for castration-resistant prostate cancer. N Engl J Med. (2010) 363:411–22. doi: 10.1056/nejmoa1001294 20818862

[20] KimJH LeeY BaeYS KimWS KimK ImHY . Phase I/II study of immunotherapy using autologous tumor lysate-pulsed dendritic cells in patients with metastatic renal cell carcinoma. Clin Immunol. (2007) 125:257–67. doi: 10.1016/j.clim.2007.07.014 17916447

[21] BapsyPP SharanB KumarC DasRP RangarajanB JainM . Open-label, multi-center, non-randomized, single-arm study to evaluate the safety and efficacy of dendritic cell immunotherapy in patients with refractory solid Malignancies, on supportive care. Cytotherapy. (2014) 16:234–44. doi: 10.1016/j.jcyt.2013.11.013 24438902

[22] JanesME GottliebAP ParkKS ZhaoZ MitragotriS . Cancer vaccines in the clinic. Bioeng Transl Med. (2023) 9:e10588. doi: 10.1002/btm2.10588 38193112 PMC10771564

[23] van WilligenWW BloemendalM GerritsenWR SchreibeltG de VriesIJM BolKF . Dendritic cell cancer therapy: vaccinating the right patient at the right time. Front Immunol. (2018) 9:2265. doi: 10.3389/fimmu.2018.02265 30327656 PMC6174277

[24] AmanyaSB Oyewole-SaidD ErnsteKJ BishtN MurthyA Vazquez-PerezJ . The mARS complex: a critical mediator of immune regulation and homeostasis. Front Immunol. (2024) 15:1423510. doi: 10.3389/fimmu.2024.1423510 38975338 PMC11224427

[25] HalpertM KonduriV LiangD Vazquez-PerezJ HofferekC WeldonS . MHC class I and II peptide homology regulates the cellular immune response. FASEB J. (2020) 34(6):8082–8101. doi: 10.1096/fj.201903002R 32298026 PMC7493300

[26] ZhouZ SunB HuangS YuD ZhangX . Roles of aminoacyl-tRNA synthetase-interacting multi-functional proteins in physiology and cancer. Cell Death Dis. (2020) 11:579. doi: 10.1038/s41419-020-02794-2 32709848 PMC7382500

[27] DiasJ RenaultL PérezJ MirandeM . Small-angle X-ray solution scattering study of the multi-aminoacyl-tRNA synthetase complex reveals an elongated and multi-armed particle. J Biol Chem. (2013) 288:23979–89. doi: 10.1074/jbc.M113.489922 23836901 PMC3745343

[28] DeckerWK XingD LiS RobinsonSN YangH SteinerD . Th-1 polarization is regulated by dendritic-cell comparison of MHC class I and class II antigens. Blood. (2009) 113:4213–23. doi: 10.1182/blood-2008-10-185470 19171878 PMC2676083

[29] LiangD TianL YouR HalpertMM KonduriV BaigYC . AIMp1 potentiates T_H_1 polarization and is critical for effective antitumor and antiviral immunity. Front Immunol. (2018) 8:1801. doi: 10.3389/fimmu.2017.01801 29379495 PMC5775236

[30] KonduriV JosephSK ByrdTT NawasZ Vazquez-PerezJ HofferekCJ . A subset of cytotoxic effector memory T cells enhances CAR T cell efficacy in a model of pancreatic ductal adenocarcinoma. Sci Transl Med. (2021) 13:eabc3196. doi: 10.1126/scitranslmed.abc3196 33952672 PMC10316998

[31] KonduriV Oyewole-SaidD Vazquez-PerezJ WeldonSA HalpertMM LevittJM . CD8+CD161+ T-cells: cytotoxic memory cells with high therapeutic potential. Front Immunol. (2021) 11:613204. doi: 10.3389/fimmu.2020.613204 33597948 PMC7882609

[32] Georges J Clay CM Amin S Ifrach J Burns B Trivedi A . Vaccination by homologous antigenic loading with DOC1021 as adjuvant therapy for glioblastoma: phase I clinical trial results. J Clin Oncol. (2025) 43:2014. doi: 10.1200/JCO.2025.43.16_suppl.2014

[33] Slagter-JägerJG RaneyA LewisWE DebenedetteMA NicoletteCA TcherepanovaIY . Evaluation of RNA amplification methods to improve DC immunotherapy antigen presentation and immune response. Mol Ther Nucleic Acids. (2013) 2:e91. doi: 10.1038/mtna.2013.18 23653155 PMC4817939

[34] DeckerWK XingD LiS RobinsonSN YangH YaoX . Double loading of dendritic cell MHC class I and MHC class II with an AML antigen repertoire enhances correlates of T-cell immunity *in vitro* via amplification of T-cell help. Vaccine. (2006) 24:3203–16. doi: 10.1016/j.vaccine.2006.01.029 16480795

[35] HalpertMM KonduriV LiangD ChenY WingJB PaustS . Dendritic cell-secreted cytotoxic T-lymphocyte-associated protein-4 regulates the T-cell response by downmodulating bystander surface B7. Stem Cells Dev. (2016) 25:774–87. doi: 10.1089/scd.2016.0009 26979751 PMC4870609

[36] KonduriV HalpertMM YunyuC CoronadoR RodgersJR LevittJ . Dendritic cell vaccination plus low-dose doxorubicin for the treatment of spontaneous canine hemangiosarcoma. Cancer Gene Ther. (2019) 26:282–91. doi: 10.1038/s41417-019-0080-3 30670791 PMC6760631

[37] RosinRD HaynesA KiddM DrozdovI ModlinI HalimA . Evaluation of a multigenomic liquid biopsy (PROSTest) for prostate cancer detection and follow-up in a Caribbean population. Cancer Epidemiol. (2024) 92:102642. doi: 10.1016/j.canep.2024.102642 39121520

[38] RahbarK KiddM PrasadV David RosinR DrozdovI HalimA . Clinical sensitivity and specificity of the PROSTest in an American cohort. Prostate. (2025) 85:558–66. doi: 10.1002/pros.24858 39838708 PMC11934832

[39] ZhanL FengHF LiuHQ GuoLT ChenC YaoXL . Immune checkpoint inhibitors-related thyroid dysfunction: epidemiology, clinical presentation, possible pathogenesis, and management. Front Endocrinol (Lausanne). (2021) 12:649863. doi: 10.3389/fendo.2021.649863 34177799 PMC8224170

[40] AnichiniA RaggiD BrigantiA MassaS LucianoR MaurizioC . Pembrolizumab as neoadjuvant therapy before radical cystectomy in patients with muscle-invasive urothelial bladder carcinoma (PURE-01): an open-label, single-arm, phase II study. J Clin Oncol. (2018) 36:3353–60. doi: 10.1200/JCO.18.01148 30343614

[41] SharmaP Hu-LieskovanS WargoJA RibasA . Primary, adaptive, and acquired resistance to cancer immunotherapy. Cell. (2017) 168:707–23. doi: 10.1016/j.cell.2017.01.017 28187290 PMC5391692

[42] ChenJ MaN ChenB HuangY LiJ LiJ . Synergistic effects of immunotherapy and adjunctive therapies in prostate cancer management. Crit Rev Oncology/Hematology. (2024) 207:104604. doi: 10.1016/j.critrevonc.2024.104604 39732304

[43] SchmidP AdamsS RugoHS SchneeweissA BarriosCH IwataH . Atezolizumab and nab-paclitaxel in advanced triple-negative breast cancer. N Engl J Med. (2018) 379:2108–21. doi: 10.1056/NEJMoa1809615 30345906

[44] Díaz-TejedorA Lorenzo-MohamedM PuigN García-SanzR MateosMV GarayoaM . Immune system alterations in multiple myeloma: molecular mechanisms and therapeutic strategies to reverse immunosuppression. Cancers (Basel). (2021) 13:1353. doi: 10.3390/cancers13061353 33802806 PMC8002455

[45] KlusaD LohausF FuresiG RaunerM BenešováM KrauseM . Metastatic spread in prostate cancer patients influencing radiotherapy response. Front Oncol. (2021) 10:627379. doi: 10.3389/fonc.2020.627379 33747899 PMC7971112

[46] SaxenaM BhardwajN . Re-emergence of dendritic cell vaccines for cancer treatment. Trends Cancer. (2018) 4:119–37. doi: 10.1016/j.trecan.2017.12.007 29458962 PMC5823288

[47] JungNC LeeJH ChungKH KwakYS LimDS . Dendritic cell-based immunotherapy for solid tumors. Transl Oncol. (2018) 11:686–90. doi: 10.1016/j.tranon.2018.03.007 29627706 PMC6154348

[48] BurnsBA ChandraM KonduriV DeckerWK . High tumor CD161 expression predicts a survival advantage and marks a Th1-skewed microenvironment. Front Immunol. (2025) 16:1522755. doi: 10.3389/fimmu.2025.1522755 40165951 PMC11955640

[49] SantiniSM LapentaC LogozziM ParlatoS SpadaM Di PucchioT . Type I interferon as a powerful adjuvant for monocyte-derived dendritic cell development and activity *in vitro* and in Hu-PBL-SCID mice. J Exp Med. (2000) 191:1777–88. doi: 10.1084/jem.191.10.1777 10811870 PMC2193160

