# Supplementary material for: Case Report: Primed dendritic cell immunotherapy for multiple solid tumors
Source: Front Immunol. 2026 Aug 11;17:1886898. doi: 10.3389/fimmu.2026.1886898 (PMC13503334; doi:10.3389/fimmu.2026.1886898)
Supplement: Supplementary file 1 [file Table1.docx]

# Supplemental Materials

| **DC Identity Markers:** | **Prostate Adenocarcinoma** | **Malignant Melanoma** | **Desmoplastic Melanoma** | **TNBC** | **Urothelial Carcinoma** |
| --- | --- | --- | --- | --- | --- |
| CD45+ | 99.94% | 88.42% | 99.43% | 99.45% | 94.84% |
| Viability (7-ADD) | 99.97% | 73.69% | 85.94% | 99.84% | 93.30% |
| CD83+ | 96.82% | 96.03% | 99.06% | 70.55% | 81.33% |
| CD86+ | 97.60% | 6.05% | 12.28% | 92.01% | 98.73% |
| HLA-DR | 99.16% | 85.02% | 98.46% | 98.29% | 98.73% |
| CD14 | 99.71% | 0.17% | 0.42% | 17.66% | 79.54% |
| CD3 | 0% | 0.31% | 0.97% | 0% | 0.53% |
| CD11c | 99.98% | 98.57% | 90.14% | 97.30% | 88.32% |
| CD80 | 99.90% | 99.43% | 86.36% | 99.76% | 66.45% |
| CD83+CD11c+ | 98.07% | 92.19% | 91.56% | 81.51% | 88.39% |
| CTLA-4 (intracellular) | 99.95% | 99.8% | 90.13% | 99.64% | 95.40% |
| CD14+HLA-DR+ | 98.99% | N/A | N/A | 44.25% | 79.05% |

1. **Specific Dendritic Cell Identity and Viability Markers for the Individual Cases.**

|  | **PCa** | **Malignant Melanoma** | **Desmoplastic Melanoma** | **TNBC** | **Urothelial Carcinoma** |
| --- | --- | --- | --- | --- | --- |
| Number of Monocytes collected (billions) | 22.5 | 6.9 | 27.6 | 0.8 | 24.9 |
| Number of primed DC generated (millions) | 40.4 | 173.6 | 250 | 52.4 | 20.2 |
| Dosage of cells per injection (millions) | 1. 7.0  2. 4.56  3. 2.24 | 1. 4.34  2. 12.5  3. 12.52 | 1. 8.1  2. 20.6  3. 17.6 | 1. 7.0  2. 3.56  3. 6.3 | 1. 7.0  2. 1.5  3. 2.3 |
| Pegylated interferon-alpha | 1. 180 mcg Left Deltoid  2. 180 mcg Right Deltoid  3. 180 mcg Left Deltoid | 1. 180 mcg Left Deltoid  2. 180 mcg Right Deltoid  3. 180 mcg Left Deltoid | 1. 180 mcg Left Deltoid  2. 180 mcg Right Deltoid  3. 180 mcg Left Deltoid | 1. 180 mcg Left Deltoid  2. 180 mcg Right Deltoid  3. 180 mcg Left Deltoid | 1. 180 mcg Left Deltoid  2. 180 mcg Right Deltoid  3. 180 mcg Left Deltoid |

2. **Additional Information about the DC treatment.**
